# Diagnostic Value of Imaging Combined With Tumor Markers in Early Detection of Lung Cancer

**DOI:** 10.3389/fsurg.2021.694210

**Published:** 2021-11-26

**Authors:** Su-Ju Wei, Li-Ping Wang, Jun-Yan Wang, Jing-Xu Ma, Feng-Bin Chuan, Yu-Dong Zhang

**Affiliations:** ^1^Department of Medical Oncology, The Fourth Hospital of Hebei Medical University, Shijiazhuang, China; ^2^Department of Oncology, Baotou Central Hospital, Baotou, China; ^3^Department of Imaging, The Second Affiliated Hospital of Xinjiang Medical University, Urumqi, China; ^4^Department of Respiratory and Critical Care Medicine, Weinan Central Hospital, Weinan, China; ^5^Department of Thoracic Surgery, Affiliated Hospital of Nantong University, Nantong, China

**Keywords:** lung cancer, imaging, tumor markers, diagnostic value, CEA

## Abstract

**Objective:** The objective of this research is to explore the diagnostic value of imaging plus tumor markers in the early detection of lung cancer.

**Methods:** Sixty patients with lung cancer treated in our hospital from January 2018 to January 2019 were selected as group A. They were matched with 60 patients with benign lung disease as group B and 60 healthy subjects examined in our hospital as group C. The carcino-embryonic antigen (CEA), CYFRA21-1, and neuron-specific enolase (NSE) were assessed, and the diagnostic value of tumor markers plus imaging in lung cancer diagnosis was explored.

**Results:** The CEA, CYFRA21-1, and NSE in group A were evidently superior to those in groups B and C, and those in group B were superior to those in group C (all *P* < 0.001). CEA had the highest sensitivity (56.7%), and NSE had the highest specificity (93.3%). The tumor markers plus imaging had the highest sensitivity for different types of lung cancer, and the sensitivity to early lung cancer (90%) was superior to other diagnostic methods (*P* < 0.05).

**Conclusion:** The tumor markers plus imaging is of great significance in early lung cancer diagnosis and provides a reference for judging the pathological classification.

## Introduction

Lung cancer, one of the most lethal malignancies in clinical practice, lacks the specificity of early symptoms, resulting in difficulty in diagnosis. Many patients are in the middle or advanced stages when diagnosed, normally miss the optimal treatment timing, thus increasing the mortality risk. Thus, the development of an early diagnostic approach of lung cancer to improve the survival rate is highly desirable. As one of the routine detection methods for lung cancer, tumor markers are convenient and easy to obtain but with poor sensitivity. Therefore, it is necessary to combine multiple tumor markers together to improve the diagnosis rate. With the development of multi-slice spiral CT technology, it has been widely used in practice. With the merit of being clear, simple, and efficient, it has played a certain positive role in early lung cancer diagnosis (1–4). However, studies that are concerned about the combination with CT and tumor markers are scant. Accordingly, a total of 180 eligible patients were enrolled to explore the diagnostic value of combined diagnosis, and the results are as follows. The present study is unique in the sense that multiple indexes were involved in the detection, which has guiding significance for the early diagnosis of lung cancer.

## Materials and Methodology

### General Information

Sixty patients with lung cancer treated in our hospital from January 2018 to January 2019 were selected as group A. They were matched with 60 patients with benign lung disease as group B and 60 healthy subjects examined in our hospital as group C. The ethical committee of the hospital has approved the study. Comparison of the smoking history rate of the three groups of patients was statistically significant (*P* < 0.05); whereas other general data among the three groups were similar (*P* > 0.05), as shown in Table 1.

**Table 1:** The general data of the three groups.

	**Group A** ** (*n =* 60)**	**Group B** ** (*n =* 60)**	**Healthy group** ** (*n =* 60)**	***P***
Gender (male/female)	42/18	41/19	40/20	>0.05
Average age	51.21 ± 6.20	51.23 ± 6.21	51.25 ± 5.89	>0.05
Income (yuan)				>0.05
<3,000	25	26	27	
≥3,000	35	34	33	
Literacy levels				
High school and below	21	22	20	>0.05
University and above	39	38	40	
Alcohol drinking	32	30	29	>0.05
Overweight	4	5	2	>0.05
History of smoking	56	20	10	0.001

### Inclusion Criteria

The inclusion criteria are as follows: (a) patients and their families who are informed of the purpose and process of this experiment, and a consent form had been signed; (b) with complete clinical data; (c) diagnosed with lung cancer in group A, while excluded from the possibility of lung cancer in group B.

### Exclusion Criteria

The exclusion criteria are as follows: (a) patients with other organ diseases and chronic diseases; (b) with mental problems or unable to communicate; (c) with a history of malignancy; (d) related to any other participants.

### Method

#### Imaging Diagnosis

The subjects were informed to inhale and hold their breath; the pre-scan method was used to obtain the best level to show the lesions in the patient; the multi-slice spiral CT machine (Philips Brilliance 16-slice spiral CT instrument, SFDA certified no. 2009 No. 3300931, Amsterdam, The Netherlands) was set to 120 kV, 130 mA; the layer thickness and the layer distance were both 10 mm; and the thread pitch was 1. An appropriate window width and position were chosen for observation to make the diseased tissue clearer and more intuitive. Three experienced doctors were asked to read the image, and the final results were defined by all doctors.

#### Tumor Markers

In the morning, 5 ml of fasting peripheral venous blood was taken. The serum levels of carcino-embryonic antigen (CEA), CYFRA21-1, and neuron-specific enolase (NSE) were determined by electro-chemiluminescence immunoassay (COBASE 411, original matching reagent, SFDA Certified No. 3402843). The electrolysis reaction was carried out by applying a certain waveform voltage or current signal on the electrode so that the coexisting components in the system react to produce chemiluminescence phenomenon to quantify/qualify the tumor marker to be tested. The reference ranges were ≤ 5, ≤ 3.3, and ≤ 16.3 ng/ ml, respectively.

### Observation Indexes

The serum levels of CEA, CYFRA21-1, and NSE of all groups were assessed.

#### Diagnostic Value of Imaging Plus Tumor Markers

The following indexes were compared. (a) Sensitivity: (positive cases in group A)/(total number in group A); (b) specificity: (negative cases in group B + negative cases in the group C)/(the total number of group B + the total number of group C); (c) positive predictive value: (positive cases in the group A)/(positive cases in group A + positive cases in group B + positive cases in group C); (d) negative predictive value: (negative cases in the group A)/(negative cases in group A + negative cases in group B + negative cases in group C) (5–8).

#### Classification of Lung Cancer

The lung cancer was divided into squamous cell carcinoma, adenocarcinoma, small cell lung cancer, and mixed lung cancer. The number and proportion of each category were calculated.

#### Sensitivity and Diagnostic Value of Combined Diagnosis

The combined diagnosis was conducted to determine the sensitivity of different combined diagnosis methods for different types of lung cancer. Then, the sensitivity of combined diagnosis for early lung cancer was calculated.

### Statistical Methods

The data were processed by the SPSS22.0 statistical software, and the graphics were plotted by GraphPad prism 8.0. Measurement data were expressed as ($\bar{x}\;\pm$ s) and tested by one-way ANOVA. The receiver operating characteristic (ROC) curve was used to determine the area under curve (AUC); *P* < 0.05 indicated the statistical difference.

## Results

### Comparison of Tumor Markers in Three Groups

**Figure 1:**
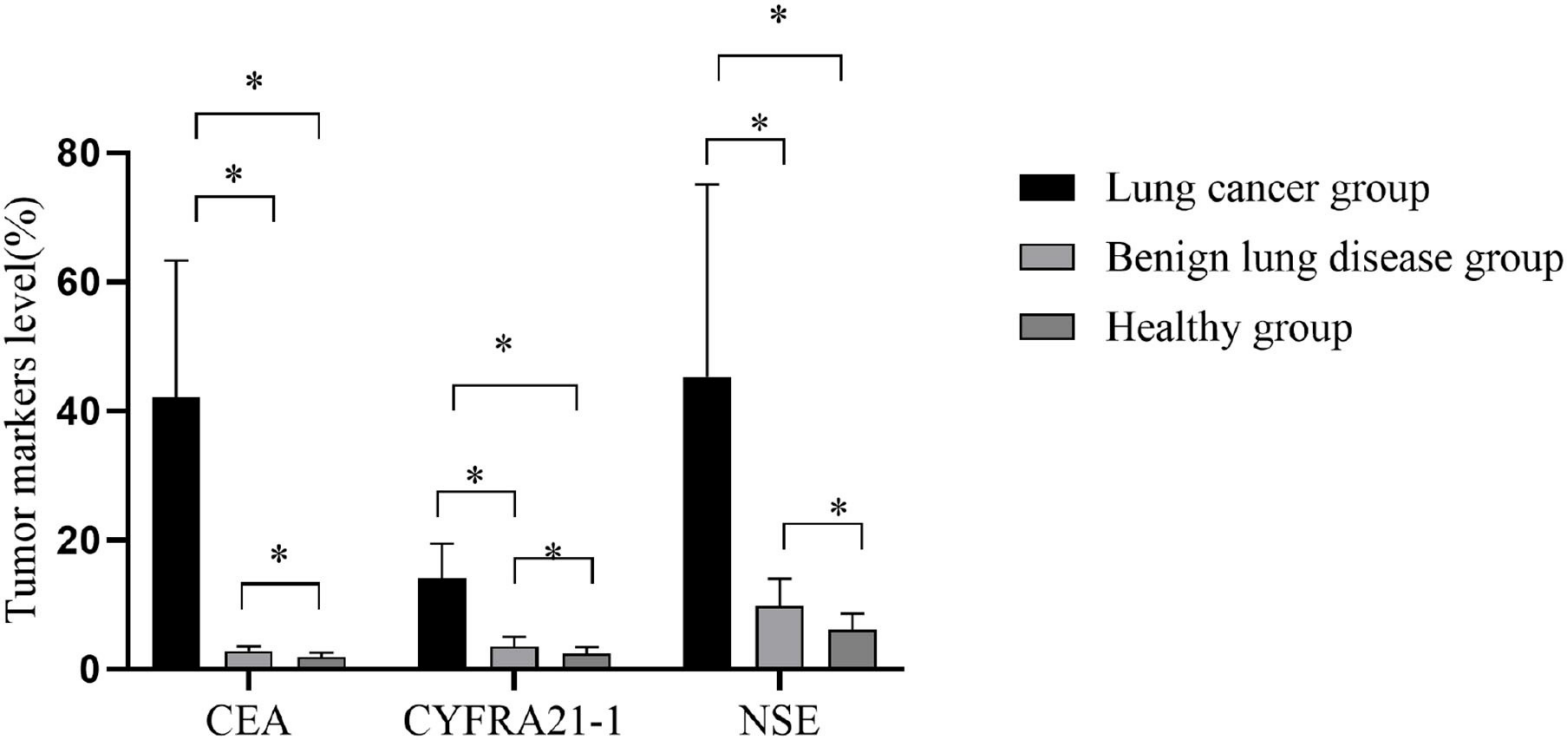
Comparison of tumor markers in three groups. The horizontal axis from left to right were carcino-embryonic antigen (CEA), CYFRA21-1, and neuron-specific enolase (NSE). The CEAs of group A, group B, and group C were 42.1 ± 21.2, 2.8 ± 0.8, and 1.9 ± 0.7 ng/ml, respectively. The CYFRA21-1 were 14.1 ± 5.3, 3.5 ± 1.5, and 2.4 ± 1 ng/ml, respectively. The NSEs were 45.3 ± 29.8, 9.8 ± 4.2, and 6.1 ± 2.5 ng/ml, respectively. *P < 0.001.

The CEA, CYFRA21-1, and NSE in group A were considerably superior to those in groups B and C, and those in group B were superior to those in group C (all *P* < 0.001), as shown in Figure 1.

### Comparison of the Diagnostic Value in Tumor Markers and Imaging Diagnosis

In the early diagnosis of lung cancer, CEA had the highest sensitivity (56.7%) and NSE had the highest specificity (93.3%), as shown in Table 2.

**Table 2:** Comparison of tumor markers in three groups.

	**Sensitivity (%)**	**Specificity (%)**	**Positive predictive value (%)**	**Negative predictive value (%)**
CEA	56.7 (34/60)	91.7 (110/120)	77.3 (34/44)	80.9 (110/136)
NSE	33.3 (20/60)	93.3 (112/120)	71.4 (20/28)	73.7 (112/152)
CYFRA21-1	46.7 (28/60)	92.5 (111/120)	75.7 (28/37)	77.6 (111/143)
Imaging diagnosis	50.0 (30/60)	87.5 (105/120)	66.7 (30/45)	77.8 (105/135)

### Classification Statistics of Lung Cancer

**Figure 2:**
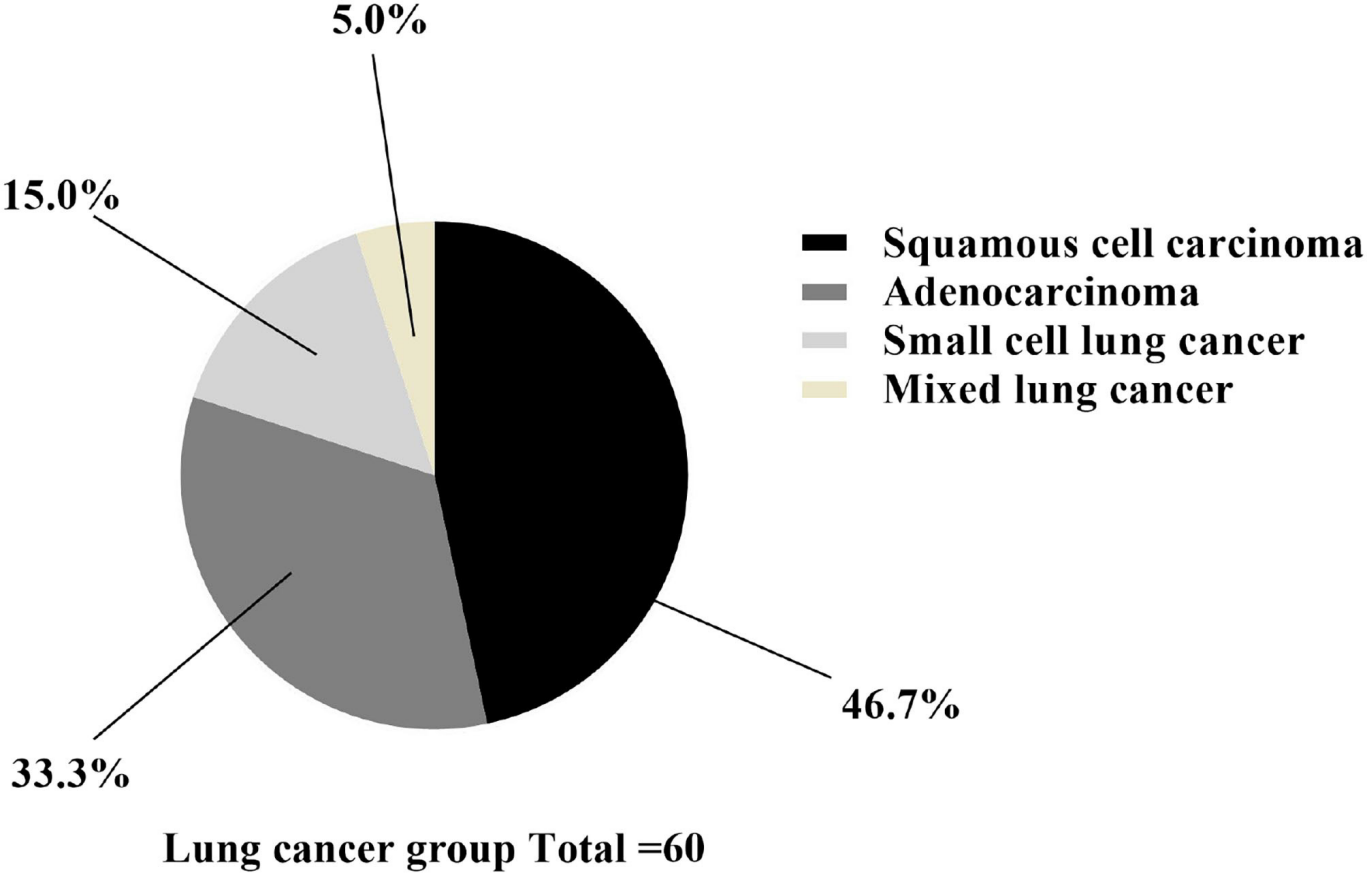
Classification statistics of lung cancer. The black area was squamous cell carcinoma (*n* = 28), the dark gray area was adenocarcinoma (*n* = 20), the light gray area was small cell lung cancer (*n* = 9), and the yellow area was mixed lung cancer (*n* = 3).

In group A, 28 patients had squamous cell carcinoma, 20 had adenocarcinoma, 9 had small cell lung cancer, and 3 had mixed lung cancer, as shown in Figure 2.

### Comparison of Sensitivity and Specificity in the Combined Diagnosis

**Figure 3:**
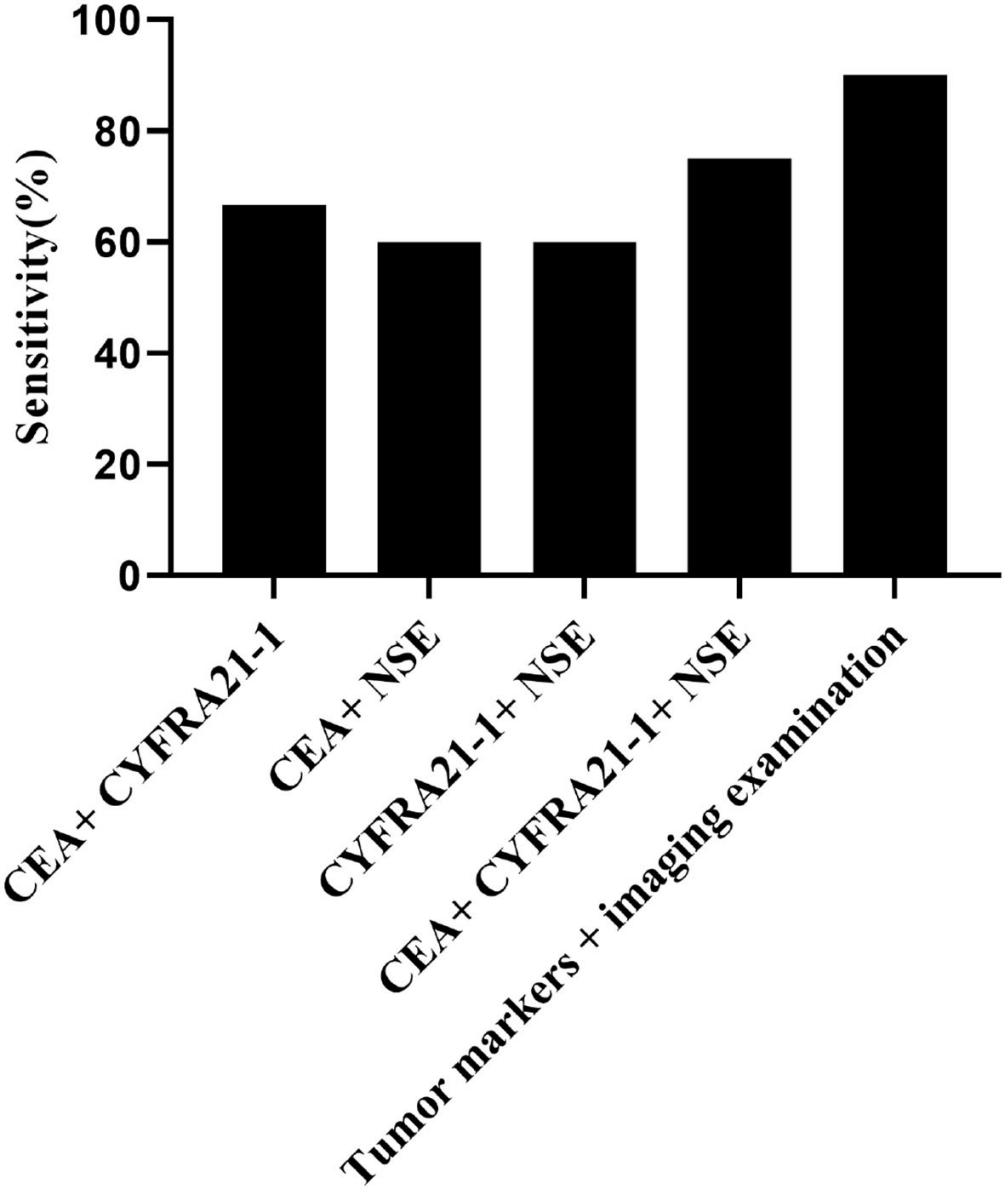
Comparison of sensitivity in the combined diagnosis. The horizontal axis from left to right were CEA + CYFRA21-1, CEA + NSE, CYFRA21-1 + NSE, CEA + CYFRA21-1 + NSE, and tumor markers combined with imaging diagnosis. The sensitivities of above methods were 66.7% (40/60), 60% (36/60), 60% (36/60),75% (45/60), and 90% (54/60), respectively.

The imaging diagnosis combined with tumor markers had the highest sensitivity of different types of lung cancer, and the sensitivity to lung cancer (90%) was superior to other diagnostic methods (*P* < 0.05), as shown in Tables 3, 4 and Figure 3.

**Table 3:** Comparison of sensitivity in the combined diagnosis.

	**Squamous cell carcinoma (*n =* 28)**	**Adenocarcinoma (*n =* 20)**	**Small cell lung cancer (*n =* 9)**	**Mixed lung cancer (*n =* 3)**
CEA+ CYFRA21-1	21 (75.0)	16 (80.0)	2 (22.2)	1 (33.3)
CEA+ NSE	19 (67.9)	12 (60.0)	4 (44.4)	1 (33.3)
CYFRA21-1+ NSE	20 (71.4)	11 (55.0)	4 (44.4)	1 (33.3)
CEA+ CYFRA21-1+ NSE	23 (82.1)	16 (80.0)	5 (55.6)	1 (33.3)
Tumor markers combined with Imaging Diagnosis	26 (92.9)	18 (90.0)	7 (77.8)	3 (100.0)

**Table 4:** Comparison of specificity in the combined diagnosis.

	**Squamous cell carcinoma (*n =* 28)**	**Adenocarcinoma (*n =* 20)**	**Small cell lung cancer (*n =* 9)**	**Mixed lung cancer (*n =* 3)**
CEA+ CYFRA21-1	22 (78.6)	17 (85.0)	3 (33.3)	1 (33.3)
CEA+ NSE	20 (71.4)	13 (65.0)	4 (44.4)	1 (33.3)
CYFRA21-1+ NSE	20 (71.4)	12 (60.0)	4 (44.4)	1 (33.3)
CEA+ CYFRA21-1+ NSE	24 (85.7)	16 (80.0)	5 (55.6)	2 (66.7)
Tumor markers combined with Imaging Diagnosis	27 (96.4)	19 (95.0)	8 (88.9)	3 (100.0)

### Diagnostic Value

**Figure 4:**
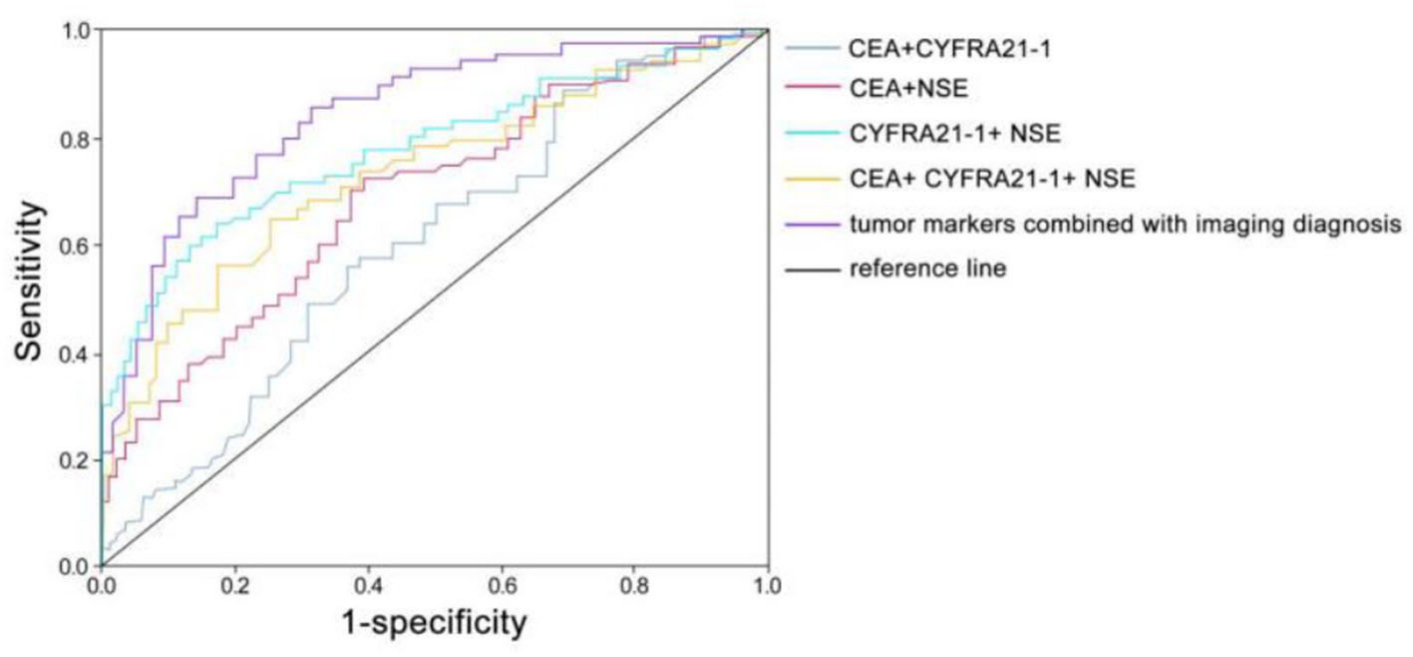
Receiver operating characteristic (ROC) curve diagnostic value.

According to the ROC curve, when the AUC of CEA+CYFRA21-1 is 0.653 (95% CI: 0.578–0.728), the sensitivity is 0.448 and the specificity is 0.800; when the AUC of CEA+NSE is 0.699 (95% CI: 0.628–0.770), the sensitivity is 0.686 and the specificity is 0.660; when the AUC of CYFRA21-1+ NSE is 0.782 (95% CI: 0.719–0.846), the sensitivity is 0.638 and the specificity is 0.870; when the AUC of CEA+ CYFRA21-1+ NSE is 0.714 (95% CI: 0.643–0.785), the sensitivity is 0.552 and the specificity is 0.820; when the AUC of tumor markers combined with imaging diagnosis is 0.824 (95% CI: 0.766–0.882), the sensitivity is 0.857 and the specificity is 0.700 (Figure 4).

## Discussion

Carcino-embryonic antigen, a non-specific tumor marker, shows an upward trend in both malignant tumor and benign disease. Therefore, group A had a higher CEA than the other two groups, whereas group B had a higher CEA than group C (*P* < 0.001). Additionally, CEA alone had the highest sensitivity (56.7%), confirming its certain value in the early diagnosis, as shown in this study. NSE, the main tumor marker of small cell lung cancer, was significantly higher in group A than the other two groups (*P* < 0.001), with the highest specificity of NSE in group A (93.3%), indicating that NSE has a high application value in combined detection. CYFRA21-1, usually located in tumor cells of epithelial origin in the lung, is widely secreted when tumor cells were dissolved (9–12). Moreover, group A had a higher CYFRA21-1 than the other two groups (*P* < 0.001), with a higher positive predictive value than those of NSE and imaging diagnosis, suggesting a vital role of CYFRA21-1 in early diagnosis. In this study, the three tumor markers, CEA, CYFRA21-1, and NSE, are all substances that have important guiding significance for the diagnosis of lung cancer. Since a single test has certain limitations, false positive or false negative results may occur, which may mislead the clinical diagnosis. Therefore, a combined test is needed to improve the accuracy of the diagnosis of lung cancer. The combined detection of tumor markers can effectively improve the sensitivity of lung cancer diagnosis and is of great significance for the early diagnosis of lung cancer.

Lung cancer is a disease with many pathological types that single tumor marker detection cannot meet the need for an accurate diagnosis. Clinically, a variety of highly sensitive tumor markers are usually combined to accelerate the early diagnosis, and CEA, CYFRA21-1, and NSE are frequently used (13–16). It can be concluded from the comparison of the sensitivity of CEA, CYFRA21-1, and NSE alone and combined detection that a single tumor marker for diagnosis is not satisfactory, whereas the combined detection yields an excellent result in the early diagnosis. In addition, different combination methods have an impact on the detection rate of different lung cancer types. In small cell lung cancer, NSE plus other tumor markers can improve the detection rate, which is possible because small cell lung cancer has the characteristics of neuroendocrine cells. Tumor markers combined with imaging diagnosis have the highest sensitivity in the distinguishing lung cancer types, and its sensitivity for lung cancer (90%) is highly superior to other diagnostic methods (*P* < 0.05). Duncan Sullivan carried out research to compare the sensitivity of tumor marker alone and combined with imaging diagnosis and found that the sensitivity of imaging diagnosis plus CEA, CYFRA21-1, and NSE was 91.3% (73/80), confirming the high diagnostic value of the combination diagnosis in the early diagnosis (17), which is in line with this study. A number of studies have carried out different screening techniques and used screening tumor markers to predict early lung cancer, and all have achieved good results. The study conducted by Stefan-van et al. (18) is different from the current study; they synthesized phthalocyanine BODIPY dye (BODIPY = boron dipyrrolidine) [phthalocyanine-BODIPY dye (BODIPY = boron dipyrromethene)] and used it for molecular recognition of lung cancer biomarkers cyfra21-1. And the detection of CYFRA21-1 level is of great significance for the diagnosis, curative effect evaluation, and prognosis monitoring of non-small cell lung cancer, because it is considered that CEA and squamous cell carcinoma antigen (scc) as biochemical indicators generates a poor sensitivity and specificity. However, this study has improved the sensitivity and specificity by using multiple indicator–combined detection. Stefan-van et al. (19) used nanostructured materials based on Cu and Ni films deposited on textile materials (veil) and gold nanostructured microspheres for the design of new stochastic sensors. The random sensor can simultaneously detect a set of biomarkers, including epidermal growth factor receptor, neuron-specific enolase, and carcinoembryonic antigen from whole blood samples, with high reliability and recovery rate over 97%. The random sensor has shown high sensitivity and low determination levels in the detection of the proposed biomarkers, making it possible to detect lung cancer early through rapid screening of whole blood. Comnea-Stancu et al. (20) proposed a random sensor based on maltodextrin with different glucose equivalents for the determination of three lung cancer biomarkers: neuron-specific enolase, carcinoembryonic antigen, and epidermal growth factor receptor. This sensor can simultaneously determine the content of NSE, CEA, and HER-1 in whole blood (qualitative and quantitative), with a recovery rate >97%. This screening test is also helpful for the rapid and early diagnosis of lung cancer. These three studies are aimed at the detection of tumor markers using new technologies and provide new ideas for future research. The limitation of this study is that it did not conduct multivariate regression analysis and did not explore the influencing factors of early lung cancer. In the future, the risk factors will be included, aiming to develop early intervention and prevent the occurrence of lung cancer (21–23).

In summary, both tumor marker detection and imaging diagnosis have a certain positive significance for the early diagnosis of lung cancer, while the combination has the highest value in the diagnosis and classification. In practice, it should be applied according to the actual situation.

## Data Availability Statement

The original contributions presented in the study are included in the article/supplementary files, further inquiries can be directed to the corresponding authors.

## Ethics Statement

The studies involving human participants were reviewed and approved by our hospital. The patients/participants provided their written informed consent to participate in this study.

## Author Contributions

All authors listed have made a substantial, direct, and intellectual contribution to the work and approved it for publication.

## Conflict of Interest

The authors declare that the research was conducted in the absence of any commercial or financial relationships that could be construed as a potential conflict of interest.

## Publisher's Note

All claims expressed in this article are solely those of the authors and do not necessarily represent those of their affiliated organizations, or those of the publisher, the editors and the reviewers. Any product that may be evaluated in this article, or claim that may be made by its manufacturer, is not guaranteed or endorsed by the publisher.

## References

[1] Mccutchan G Hiscock J Hood K Murchie P Neal RD Newton G. Engaging high-risk groups in early lung cancer diagnosis: a qualitative study of symptom presentation and intervention preferences among the UK's most deprived communities. BMJ Open. (2019) 9:e025902. 10.1136/bmjopen-2018-025902 31122972 PMC6538016

[2] Prout HC Barham A Bongard E Tudor-Edwards R Griffiths G Hamilton W. Patient understanding and acceptability of an early lung cancer diagnosis trial: a qualitative study. Trials. (2018) 19:419. 10.1186/s13063-018-2803-4 30075741 PMC6090834

[3] Akter MS Ahmed S. 53PPulmonary tuberculosis: a hurdle to overcome for early lung cancer diagnosis in TB burden countries. Ann Oncol. (2019) 30:II19. 10.1093/annonc/mdz070.011

[4] Abir A. An automated computer system based on genetic algorithm and fuzzy systems for lung cancer diagnosis. Int J Nonlin Sci Numerical Simul. (2018) 19:583–94. 10.1515/ijnsns-2017-0048

[5] Calabrese F Lunardi F Pezzuto F Fortarezza F Vuljan SE Marquette C. Are there new biomarkers in tissue and liquid biopsies for the early detection of non-small cell lung cancer? J Clin Med. (2019) 8:414. 10.3390/jcm8030414 30917582 PMC6463117

[6] Woodman C Vundu G George A Wilson CM. Applications and strategies in nanodiagnosis and nanotherapy in lung cancer. Semin Cancer Biol. (2020) 69:349–64. 10.1016/j.semcancer.2020.02.009 32088362

[7] Arsenev A Novikov S Barchuk A Kanaev S Barchuk A Tarkov S. Lung cancer diagnosis: non-invasive and invasive methods. Voprosy Onkologii. (2020) 66:42–9. 10.37469/0507-3758-2020-66-1-42-49

[8] Sedda G Gasparri R Spaggiari L. Lung Cancer Early Diagnosis: The Sooner, the Better. Horizons in Cancer Research. (2020)

[9] Gasparri R Sedda G Noberini R Bonaldi T Spaggiari L. Clinical application of mass spectrometry-based proteomics in lung cancer early diagnosis. Proteomics Clin Appl. (2020) 14:e1900138. 10.1002/prca.201900138 32418314

[10] Ikeda N Usuda J Maehara S. Photodynamic therapy for central-type early-stage lung cancer. Gen Thorac Cardiovasc Surg. (2019) 68:679–83. 10.1007/s11748-019-01240-1 31749069

[11] Tsay JCJ Greenberg AK Rom WN Massion PP. Preclinical biomarkers for the early detection of lung cancer-sciencedirect. IASLC Thorac Oncol. (2017) 2018:59–68.e4. 10.1016/B978-0-323-52357-8.00008-1

[12] Izumo T Terada Y Inomata M Kuse N Awano N Tone M. Impact of preoperative pathological confirmation on surgical and postoperative outcomes of lung resection for early stage lung cancer. Adv Respire Med. (2019) 87:203–8. 10.5603/ARM.a2019.0034 31476007

[13] Ceren K Okan E Orhan A Özer M. Impact of hybrid neural network on the early diagnosis of diabetic retinopathy disease from video-oculography signals. Chaos Solitons Fractals. (2018) 114:164–74. 10.1016/j.chaos.2018.06.034

[14] Shaffie A Soliman A Khalifeh HA Ghazal M Taher F Elmaghraby A. Radiomic-Based Framework for Early Diagnosis of Lung Cancer. In: IEEE 16th International Symposium on Biomedical Imaging (ISBI) IEEE (2019). 10.1109/ISBI.2019.8759540

[15] Memon NA Mirza AM Gilani S. Segmentation of Lungs From CT Scan Images for Early Diagnosis of Lung Cancer. Enformatika (2018).

[16] Beaty BT Weiner AA. Alternatives to surgery for early-stage non–small cell lung cancer. Clinics Chest Med. (2020) 41:197–210. 10.1016/j.ccm.2020.02.001 32402355

[17] Duncan Sullivan. What is the new direction of early lung cancer diagnosis? J Clin Med. (2018) 39:57s−66s. 10.1183/09031936.03.00405303 12572703

[18] Stefan-van Staden RI Comnea-Stancu IR Yanik H Göksel M Alexandru A Durmuş M. Phthalocyanine-BODIPY dye: synthesis, characterization, and utilization for pattern recognition of CYFRA 21–1 in whole blood samples. Anal Bioanal Chem. (2017) 409:6195–203. 10.1007/s00216-017-0560-y 28852796

[19] Stefan-van Staden RI Comnea-Stancu IR Surdu-Bob CC Badulescu M. Nanostructured materials detect epidermal growth factor receptor, neuron specific enolase and carcinoembryonic antigen. Nanoscale. (2015) 7:15689–94. 10.1039/C5NR04476F 26350155

[20] Comnea-Stancu IR Stefan-van Staden R-I van Staden JF Stanciu-Gavan C. Stochastic sensors based on maltodextrins for screening of whole blood for neuron specific enolase, carcinoembryonic antigen and epidermal growth factor receptor. Microsyst Tech. (2016) 22:25–29. 10.1007/s00542-015-2635-z

[21] Prabukumar M Agilandeeswari L Ganesan K. An intelligent lung cancer diagnosis system using cuckoo search optimization and support vector machine classifier. J Ambient Intell Human Comput. (2019) 10:267–93. 10.1007/s12652-017-0655-5

[22] Capuano R Catini A Paolesse R Di Natale C. Sensors for lung cancer diagnosis. J Clin Med. (2019) 8:235. 10.3390/jcm8020235 30754727 PMC6406777

[23] Marzorati D Mainardi L Sedda G Gasparri R Spaggiari L Cerveri P. A review of exhaled breath key role in lung cancer diagnosis. J Breath Res. (2019) 13:034001. 10.1088/1752-7163/ab0684 30754033

